# The Ladies' Working Guild at Leeds

**Published:** 1913-05-24

**Authors:** 


### The Ladies' Working Guild at Leeds.

### HOSPITAL FOR WOMEN AND CHILDREN, LEEDS.

THE Ladies' Working Guild recently held their fifth annual meeting. Mrs. Cohen presided in the absence of Viscountess Mountgarret. A vote of condolence in her ladyship's recent bereavement was passed on the proposition of Mrs. Croft, seconded by Mrs. Capper Johnson. The object of this Guild is to supply some of the household linen for the use of the patients in the Hospital for Women and Children, Leeds, and one of the rules of the Guild is that the articles must be of a uniform pattern and quality, and the matron provides the hon. secretary with a list of articles, specifying the kind and size. This year 420 articles were received, of value, with subscriptions, £52. The annual report, read by the hon. secretary, Mrs. Massey Richardson, revealed a very satisfactory year's work; and a vote of thanks was accorded her for her interest in the Guild.

